# A graded personalized exercise program’s effect on muscle and body fat: randomized controlled trial

**DOI:** 10.1186/s12889-025-22453-5

**Published:** 2025-04-03

**Authors:** Jung Yeon Son, Jiyun Jung, Jung Eun Son, Sang Gyu Park, Eo Jin Park, Junga Lee, Seung Don Yoo

**Affiliations:** 1 Present Address: Department of Rehabilitation Medicine, Kyung Hee University Hospital at Gangdong, Seoul, Republic of Korea; 2 Graduate School of Public Administration, Seoul National University, Seoul, Republic of Korea; 3 Department of Biostatistics, Dongguk University College of Medicine, Gyeongju, Republic of Korea; 4 Graduate School of Sport Science, Kyung Hee University, Yongin, Republic of Korea; 5 Department of Rehabilitation Medicine, Kyung Hee University College of Medicine, 26, Kyungheedae- ro, Dongdaemun-gu, Seoul, 02447 Republic of Korea

**Keywords:** Circuit-based exercise, Sarcopenia, Physical fitness, Body composition, Ultrasound, Middle aged

## Abstract

**Objectives:**

With population aging, personalized exercise programs considering clinical and demographic factors like sex, age, and physical activity level are essential; however, research on their effects remains limited. We aimed to evaluate the effectiveness of a Global Physical Activity Questionnaire-based graded personalized exercise program tailored for middle-aged adults aged 40–69 years.

**Participants:**

We enrolled 71 middle-aged adults in their 40s, 50s, and 60s (approximately 20 participants per age group) in a parallel-group randomized controlled trial.

**Intervention:**

Participants were assigned using age-stratified randomization to a treatment or control group. Participants were categorized into three levels according to weekly physical activity measured by the Global Physical Activity Questionnaire and physical activity guidelines for adults. Each participant’s grade was determined by applying equal weight adjustments for sex, age, and physical activity level, and the participants were assigned an exercise program corresponding to their grade. The exercise intervention consisted of a circuit training program alternating between aerobic and anaerobic exercises. The control group was instructed to maintain their usual physical activity levels.

**Main outcome measures:**

Changes from before to after exercise in clinical results (body composition, physical fitness, ultrasound-measured muscle/fat thickness, and biochemical data) were recorded during the 8-week exercise program and differences between pre- and post-exercise values of the groups were analyzed using the *t*-test and Wilcoxon rank-sum test.

**Results:**

Among 64 participants who had completed the program, 33 (51.5%) participated in the exercise program. The exercise program significantly increased abdominal muscle thickness (*p* < 0.01), reduced body fat percentage (*p* = 0.02) and waist circumference (*p* = 0.01), and positively affected various physical fitness indicators.

**Conclusions:**

This study demonstrated the beneficial effects of a graded personalized exercise program on muscle thickness, body fat, and physical fitness and offers key data to support early preventive exercise programs in middle-aged adults to mitigate the risk of sarcopenia in later life.

**Trial registration:**

Registered on November, 29, 2024 at cris.nih.go.kr identifier KCT0009970.

**Supplementary information:**

The online version contains supplementary material available at 10.1186/s12889-025-22453-5.

## Background

With population aging, it is becoming increasingly critical to maintain physical health, particularly for preventing conditions such as sarcopenia, which is characterized by the progressive loss of muscle mass and function [1–6]. Exercise has long been recognized as a key factor in promoting healthy aging by improving muscle mass and reducing body fat [7–12]. Furthermore, exercise represents a non-pharmacological approach for preventing and improving metabolic diseases through various programs [13–15]. Although extensive studies have explored the effects of tailored exercise programs, their clinical effects on middle-aged adults remain insufficiently understood. The majority of existing research has primarily targeted disease-specific populations, including individuals with musculoskeletal disorders [16] and cancer survivors [17], limiting the generalizability of findings to the broader middle-aged population. Also, recent studies emphasize the importance of tailored exercise interventions to individual characteristics, demonstrating that adjusting intensity and type to match each participant’s capacity enhances effectiveness [18–21]. meta-analyses of high-intensity interval training [22] have identified a critical limitation: the absence of a structured graded approach, which is essential for optimizing exercise adaptation based on individual capacity. Without appropriate progression in intensity, exercise interventions may either be too demanding for some participants, leading to poor adherence and increased risk of injury, or insufficiently challenging for others, resulting in suboptimal physiological benefits. One approach to personalizing exercise prescriptions is through the use of tools such as the Global Physical Activity Questionnaire (GPAQ), which measures the levels of vigorous and moderate physical activity performed during work and leisure activities as well as during transportation [23–27]. Engaging in physical activity during middle age is crucial, as older adults frequently face limitations in performing balance training or physical exercises recommended for preventing frailty, sarcopenia, and falls [28–33]. However, the effects of GPAQ-based exercise programs tailored to individual physical capacity remain unexplored, particularly in middle-aged populations.

This randomized controlled trial aimed to evaluate the effectiveness of a GPAQ-based graded personalized exercise program (GPEP) tailored for middle-aged adults aged 40–69 years. The 8-week program focused on enhancing muscle thickness, reducing body fat, and improving physical fitness. By addressing the need for evidence-based GPEP, this study aimed to optimize physical health outcomes and may contribute to the prevention of sarcopenia, offering valuable insights into clinical guidelines and the development of personalized exercise programs.

## Methods

### Study population

Based on a literature review [7, 9, 13, 34–36], approximately 60 participants were required from each site to demonstrate a program effect. Assuming a 20% attrition rate, we planned to enroll approximately 70 participants.

The inclusion criteria were as follows:

Aged 40–69 years without exercise limitations

Understand the study details and agree to participate

Expected to have no changes in medication during the study period

The exclusion criteria were as follows:

Systematic exercise experience within the past 6 months

Unable to perform regular physical activity or exercise because of musculoskeletal, cardiovascular, immune, or mental disorders

Planning a pregnancy, or have given birth within the past 6 months

Planning to move long distances or personal reasons that may prevent full participation during the study period

**Fig. 1:**
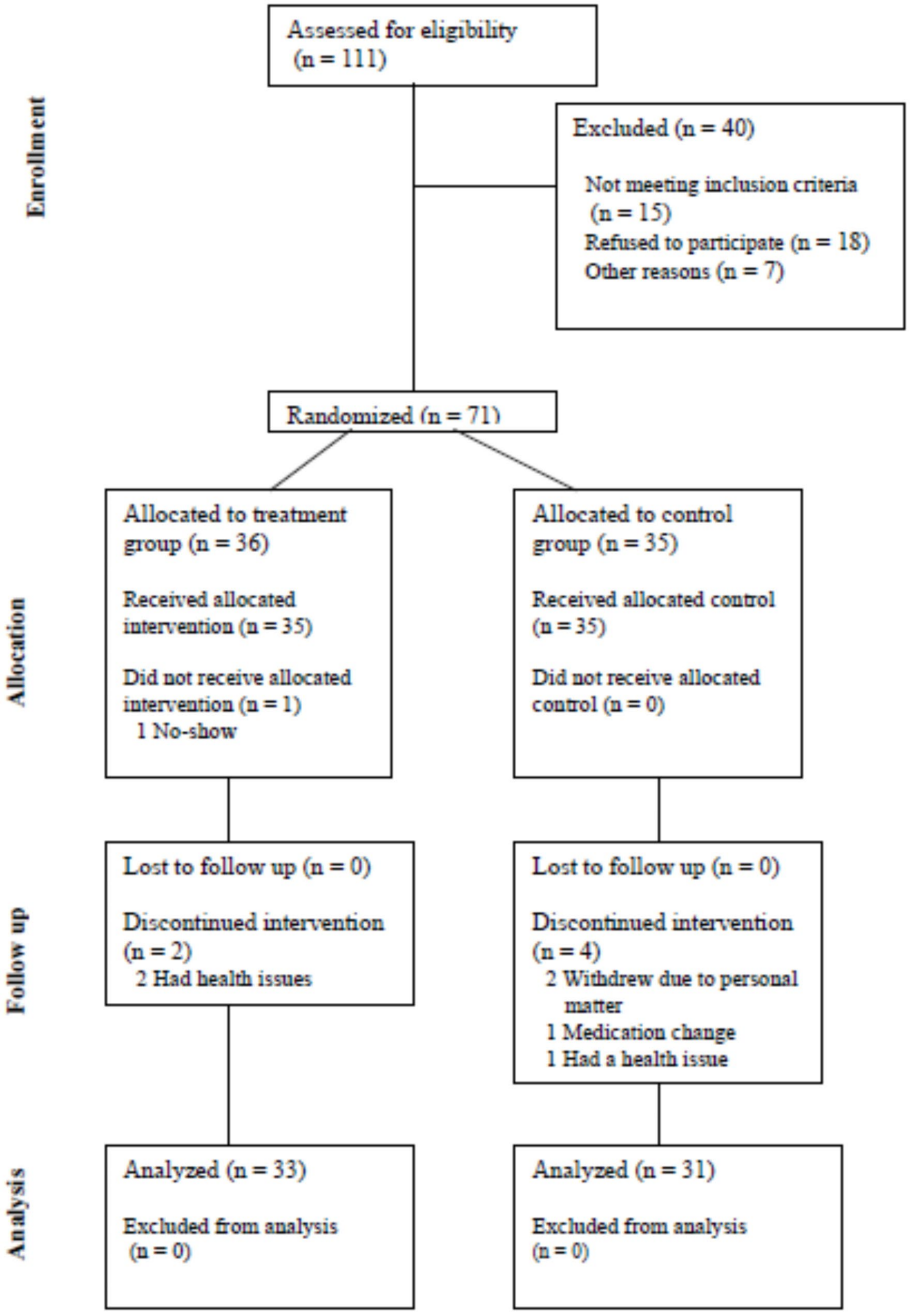
CONSORT flow diagram

This study was designed as a randomized clinical trial to assess the effects of personalized exercise programs. To validate the efficacy of the program, which was developed based on a review of previous studies [37, 38], 111 participants were assessed for eligibility, and 71 were enrolled: 24 individuals in their 40s, 24 in their 50s, and 23 in their 60s (refer to Fig. 1, the CONSORT flow diagram).The participants were stratified by age and randomly assigned to either a treatment or control group, with approximately 35 participants in each group. All data related to this study were collected using the Maven CDMS e-CRF system (JNPMEDI).

### Measurement

Before and after treatment (± 2 weeks), data were collected from the randomly assigned treatment and control groups. Body composition, height, weight, body mass index (BMI), waist circumference, lean body mass, and body fat percentage were also measured. Six physical fitness indicators were assessed: cardiorespiratory fitness (VO_2_max and step box test), muscular strength (relative grip strength), muscular endurance (cross sit-up), flexibility (sit-and-reach), agility (jumping with legs apart), and power (vertical jump). The subcutaneous fat, rectus abdominis, external oblique, internal oblique, transverse abdominis, and bilateral rectus femoris were measured using ultrasonography (HD11 Ultrasound System 795054, PHILIPS). Blood tests were conducted to collect biochemical data, including plasma lipid levels, blood pressure, HbA1c, insulin, and other relevant markers. For detailed information on the measurement methods, please refer to the additional files (Additional files 1 and 2).

### GPEP

**Fig. 2:**
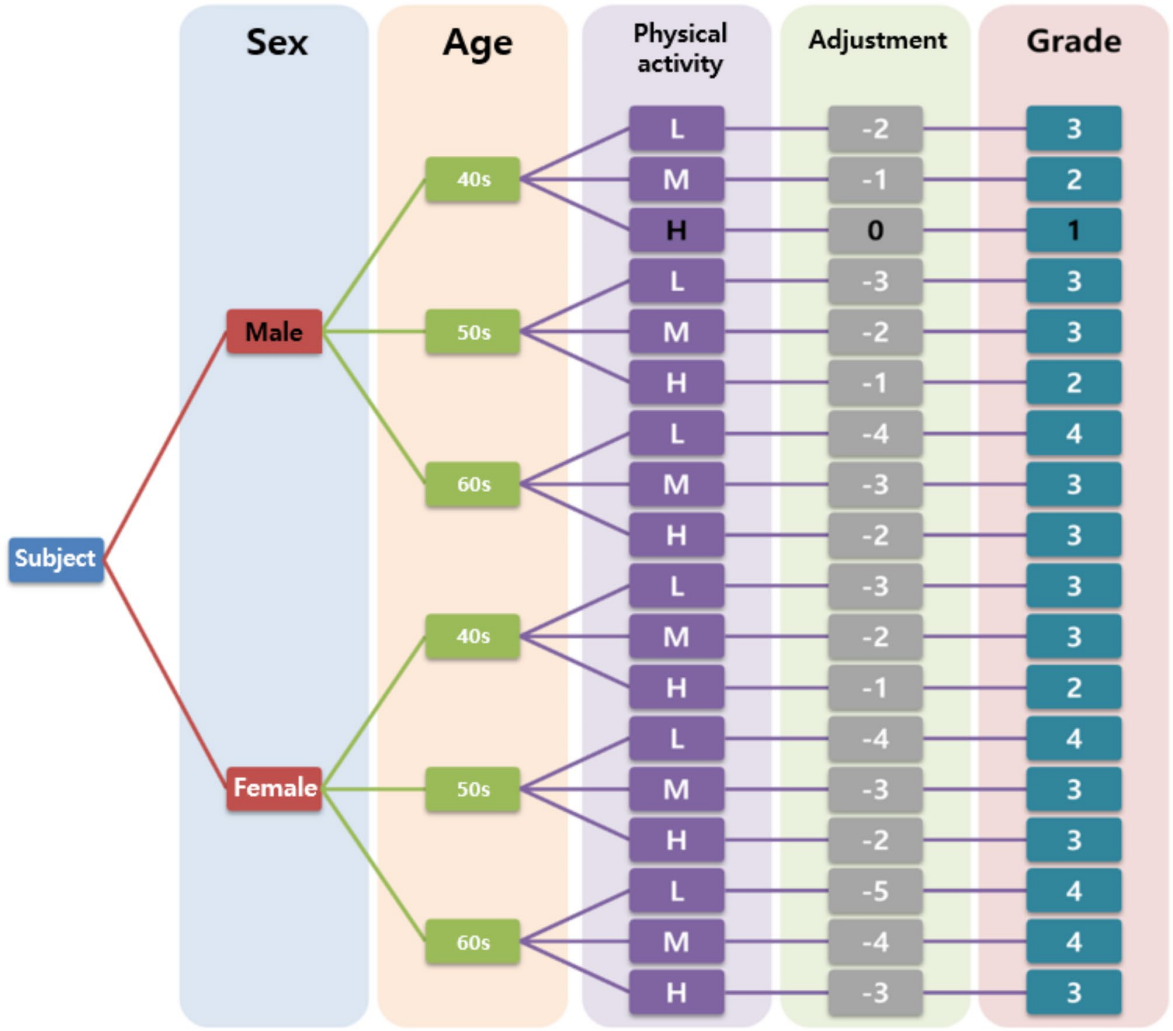
Schematic depiction of the exercise program allocation based on participant characteristics. Grade for a woman in her 60s with a low physical activity level is presented as: Score: Female (-1) + 60s (-2) + Low (-2) = -5, Grade 4

A total of 71 participants signed written informed consent forms and completed the GPAQ-modified Korean version [23], which investigates physical activity based on the type of activity and calculates the total amount of physical activity through MET^1^ values and time, reflecting intensity. Participants were categorized into three levels according to weekly physical activity measured by the GPAQ and physical activity guidelines for adults [39]: high level for more than 300 min, moderate level for 150–300 min, and low level for less than 150 min. It has been consistently reported that men tend to be more active than women and physical activity decreases with age [40, 41]. Therefore, in our study, we assumed that male participants would have higher levels of physical activity than female participants and that younger participants would be more physically active than older ones. Based on this assumption, each participant’s grade was determined by applying equal weight adjustments for sex, age, and physical activity level, and the participants were assigned an exercise program corresponding to their grade. The exercise prescription algorithm based on participant characteristics is shown in Fig. 2.

The first four weeks of the exercise program were designated as an adaptation period, allowing participants to gradually acclimate to the exercise regimen. During this period, each participant performed exercises at a lower intensity level, as determined by the algorithm developed in this study. The intensity was progressively adjusted based on individual performance and adaptation. For instance, if a 60-year-old male participant had a target intensity classified as grade 3, he would begin at a slightly lower intensity in the first week to facilitate gradual adaptation. By the end of the fourth week, his exercise intensity would be increased to reach grade 3, ensuring a smooth transition to the target level. From the fifth week onward, participants exercised at their designated target intensity, maintaining this level for the remainder of the program.

The exercise intervention consisted of a circuit training program alternating between aerobic and anaerobic exercises. Eighteen bodyweight exercises requiring no additional equipment were selected and applied based on the participants’ grades and performance abilities. The exercises included in the program were in accordance with the guidelines of the American College of Sports Medicine (ACSM) with specific exercise guidelines for each metabolic disease [42].

The 8-week program involved participants engaging in 16–24 exercise sessions, 2–3 times per week, with each session consisting of a 10-minutes warm-up, 30–40 min of circuit training, and a 5- to 10-minutes cool down. The program consisted of exercises targeting various areas, such as the upper body, lower body, full body, and core. Exercise duration gradually increased each week, whereas the rest time between sets was reduced to increase exercise intensity. Each set consisted of 12 movements, with 10 s allocated for switching movements. Considering the individual physical capacity, the movements were divided into two stages: regular movements (wide push-up, narrow push-up, burpee test, high knees, plank leg raise) and beginner movements (wide knee push-up, narrow knee push-up, slow burpee, running in place, and elbow plank leg raise; Table 1). If the participants could handle the beginner stage without difficulty, they progressed to regular movements. For example, Grade 4, with the lowest physical capacity, had a rest time of 4 min between sets; Grade 3, 3 min and 30 s; and Grade 2, 3 min, with a 30 s difference in rest time between each grade. Over intervals of 2–3 weeks, the rest time between sets gradually reduced by 30 s, whereas the exercise duration was increased by 10 s, progressively increasing the intensity of the exercise.

**Table 1:** Components of the graded personalized exercise program

Category		Type	Duration (min)
Warm-up		Stretching and aerobic exercise	10
Circuit training			
	Upper body	Wide push-up/knee wide push-up, shoulder taps, narrow push-up/knee narrow push-up	30–40
	Lower body	Squat, jumping lunge/lunge, calf raises	
	Total body	Jumping jack, burpee test/slow burpee, high knees/running in place	
	Core	Lying knee to elbows, plank leg raise/elbow plank leg raise, lying leg raises	
Cool down		Aerobic exercise	5
		Flexibility exercise	5–10

Abbreviations min: minute

In the first week, a warm-up period was set for participants to familiarize themselves with the movements and adapt to the load. During this week, only two sets were performed, with a third set added for those who completed the exercises with ease. In the second week, participants performed 10–12 repetitions within 30 s. For total-body movements, the number of repetitions varied based on execution speed: 30 repetitions for jumping jacks (one per second), 6–8 for the burpee test, 10–12 for plank leg raises, and 20 for running in place. From weeks three to five, participants exercised at a tempo of 40 beats per minute (bpm) using a metronome, aiming to maintain proper posture while completing the prescribed repetitions. Exercise duration increased from 30 to 35 s, allowing for 12 repetitions at an appropriate tempo. During this period, participants performed 35 repetitions of jumping jacks (one per second), 8–10 of the burpee test, 24 of plank leg raises, and 30 of running in place. In weeks six to eight, exercise duration further increased to 40 s, with a target of 14 repetitions. Participants completed 40 repetitions of jumping jacks (one per second), 10–12 of the burpee test, 26 of plank leg raises, and 40 of running in place. As the program progressed, those who could comfortably complete beginner-level movements individually advanced to standard movements. The transition time between movements was limited to 10 s throughout all periods. All exercises were performed in three sets. Variations in exercise intensity across different levels were achieved by adjusting the rest time between sets (Table 2).

**Table 2:** Graded exercise program by time

Circuit training		Grade 1	Grade 2	Grade 3	Grade 4
1–2 weeks	Move/set time	––––––––––––––––– 30 s/move/7.8 min/set–––––––––––––––––			
	Rest time	10 s/move 2.5 min/set	10 s/motion 3.0 min/set	10 s/move 3.5 min/set	10 s/move 4.0 min/set
3–5 weeks	Move/set time	––––––––––––––––– 35 s/move/8.8 min/set–––––––––––––––––			
	Rest time	10 s/move 2.0 min/set	10 s/move 2.5 min/set	10 s/move 3.0 min/set	10 s/move 3.5 min/set
6–8 weeks	Move/set time	––––––––––––––––– 40 s/move/9.8 min/set–––––––––––––––––			
	Rest time	10 s/move 1.5 min/set	10 s/move 2.0 min/set	10 s/move 2.5 min/set	10 s/move 3.0 min/set

Abbreviations: move, movement; s, second; min, minute

One set includes 12 motions and a 10 s rest interval

**Fig. 3:**
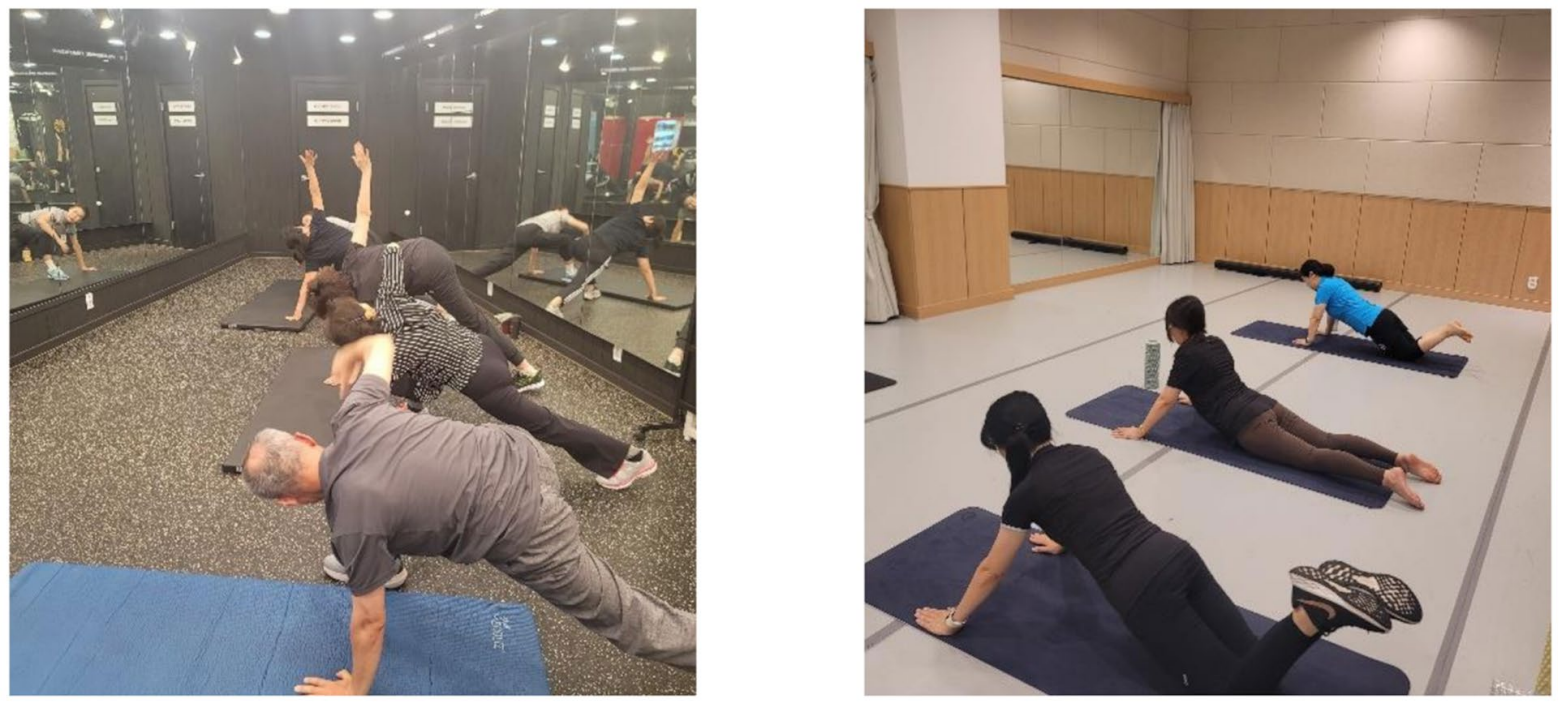
Graded personalized exercise program. (**a**) Stretching and warm-up; (**b**) Knee push-up (**a**) Participants in the treatment group performing a stretching warm-up before the circuit training. The warm-up routine was designed to enhance flexibility, improve joint mobility, and prepare participants for the main exercise program.; (**b**) Participants performing knee push-ups as part of the exercise intervention. The variation in foot positioning (raised or flat) reflects individual modifications based on participants’ physical capabilities

All participants were instructed to maintain the same meal plan as before the study. The control group included individuals without structured exercise engagement in the past six months, ensuring that they represent the general physical activity patterns of the study population. To minimize the influence of medication, any changes in the participants’ medications were monitored at each visit, and those with changes were excluded from the study. A small transportation allowance was provided for each visit. After the study concluded, restrictions on physical activity levels and medication adjustments were lifted for all groups. Figure 3 illustrates the documentation of the intervention.

### Statistical analysis

The effects of exercise were assessed by comparing the differences before and after exercise according to the intervention (control and treatment groups). To determine the appropriate method for comparing data between the groups, normality was tested using the Shapiro–Wilk test. The mean difference was evaluated using a t-test depending on the equal variance by Levene’s test when the assumption of normally distributed data was satisfied; otherwise, the Wilcoxon rank-sum test was conducted to compare the median between the groups. Based on the significant primary results, subgroup analyses according to sex and age (median, 55 years) were conducted to investigate the program’s effectiveness. All results were analyzed using R software version 4.4.2 (R Project for Statistical Computing, Vienna, Austria), and statistical significance was set at a *p*-value of < 0.05.

## Results

Table 3 summarizes the baseline characteristics of the treatment (*n* = 33) and control (*n* = 31) groups. More than half of the participants were women, and the median age was 54 years (range, 45–61 years), regardless of participation in the exercise program. Most participants were non-smokers and non-alcohol drinkers, with statistically similar characteristics between the groups.

We compared muscle and fat characteristics depending on exercise and found increased muscle thickness in the treatment group (Table 4). The abdominal muscle thickness represented by external oblique increased by 0.42 mm in the treatment group but decreased by 0.31 mm in the control group (*p* < 0.01). In addition, the thickness of the left rectus femoris increased by 0.56 mm in the treatment group and decreased by 0.59 mm in the control group, with a significant difference between the groups (*p* < 0.01) (Table 4).

**Table 3:** Descriptive characteristics of the treatment and control groups

Variable		Treatment (*n* = 33)	Control (*n* = 31)	*p*-value
Sex, n (%)				0.171
	Male	4 (12.1)	9 (29.0)	
	Female	29 (87.9)	22 (71.0)	
Age, median (range)		54.0 (45.0–61.0)	54.0 (45.5–61.0)	0.830
Smoking, n (%)				1.000
	Yes	1 (3.0)	1 (3.2)	
	No	32 (97.0)	30 (96.8)	
Drinking, n (%)				0.885
	Yes	9 (27.3)	7 (22.6)	
	No	24 (72.7)	24 (77.4)	

Our exercise program did not change BMI or lean body mass but was effective in reducing BFP and waist circumference (Table 5). The treatment group had an average decrease of 0.46% for BFP and 3.1 cm for waist circumference, whereas an average increase of 0.57% and a decrease of 0.6 cm were observed in the control group, with showing *p*-value of 0.02 and 0.01 for group difference, respectively.

**Table 4:** Intergroup differences in fat and muscle thickness using ultrasound before and after exercise

Variable			Group	Mean (SD)	Quantile				*p*-value
					0	50	100	IQR	
Fat mass									
		Subcutaneous fat	T	−1.14 (3.61)	−9.60	−0.93	10.07	3.77	0.16
			C	0.08 (3.28)	−5.40	−0.10	8.20	2.95	
Muscle mass									
		Rectus abdominis	T	0.61 (1.20)	−3.77	0.86	2.24	1.43	0.12
			C	0.44 (1.01)	−1.68	0.33	4.82	0.63	
		External oblique	T	0.42 (1.10)	−1.84	0.28	3.45	0.78	< 0.01*
			C	−0.31 (1.06)	−2.82	−0.34	2.21	0.92	
		Internal oblique	T	0.20 (1.25)	−2.69	0.00	2.78	1.59	0.49
			C	−0.04 (1.49)	−2.91	−0.03	4.59	1.71	
		Transverse abdominis	T	0.24 (0.99)	−3.74	0.27	2.57	0.62	0.36
			C	0.12 (0.89)	−1.38	0.05	2.65	1.09	
		Total abdominal muscles	T	1.47 (2.69)	−2.76	1.70	9.31	3.28	0.08
			C	0.22 (3.00)	−6.12	−0.37	8.89	2.79	
		Rt rectus femoris	T	0.08 (2.08)	−5.07	0.40	3.70	1.60	0.46
			C	−0.24 (1.39)	−3.10	−0.43	3.33	1.37	
		Lt rectus femoris	T	0.56 (1.77)	−4.80	0.66	3.73	2.33	< 0.01*
			C	−0.59 (1.55)	−3.73	−0.60	2.43	1.95	

Abbreviations: T, treatment group; C, control group; SD, standard deviation; IQR, interquartile range; Rt, right; Lt, left

^*^Statistically significant

**Table 5:** Intergroup differences in body composition indicators before and after exercise

Variable	Group	Mean (SD)	Quantile				*p*-value
			0	50	100	IQR	
BFP	T	−0.46 (3.33)	−5.40	−1.50	14.90	2.30	0.02*
	C	0.57 (3.27)	−2.70	0.20	16.30	1.95	
BMI	T	−0.15 (0.50)	−1.50	0.00	0.60	0.70	0.08
	C	0.06 (0.48)	−1.40	0.00	0.90	0.40	
LBM	T	0.34 (1.17)	−2.90	0.30	2.60	1.40	0.22
	C	−1.41 (6.51)	−34.20	0.20	2.80	1.60	
Waist Circumference	T	−3.10 (3.61)	−12.00	−3.00	3.00	5.20	0.01*
	C	−0.60 (4.16)	−11.00	0.00	10.00	5.00	

Abbreviations: T, treatment group; C, control group; SD, standard deviation; IQR, interquartile range; BFP, body fat percentage; BMI, body mass index; LBM, lean body mass

BFP (Body Fat Percentage) = (fat mass / weight (kg)) × 100, measured using INBODY S10

^*^Statistically significant

Physical fitness was assessed using the program (Table 6). We found a strengthened cross sit-up test of 8.76 reps, vertical jump by 0.04 sc, VO_2_ Max by 1.02 ml/kg/min, and sit-and-reach test by 3.81 cm in the exercise participants, and the differences were all significant. However, the program yielded minimal significant changes in blood test results (Additional file 3)

**Table 6:** Group differences in physical fitness outcomes between treatment and control groups according to before and after exercise

Variable	Group	Mean (SD)	Quantile				*p*-value
			0	50	100	IQR	
Cross sit-up test, reps	T	8.76 (6.37)	−6.00	9.00	24.00	8.00	< 0.01*
	C	0.68 (4.77)	−12.00	0.00	13.00	4.00	
Vertical jump, s	T	0.04 (0.04)	−0.02	0.03	0.12	0.04	< 0.01*
	C	0.01 (0.08)	−0.06	0.00	0.42	0.03	
Relative grip strength, %	T	3.57 (6.49)	−15.51	3.78	16.98	8.56	0.06
	C	1.55 (6.27)	−8.12	0.03	22.42	7.14	
Step box test, ml/kg/min	T	−7.48 (15.47)	−41.00	−8.00	39.00	13.00	0.10
	C	−1.45 (12.28)	−31.00	−1.00	25.00	17.00	
VO_2_ Max ml/kg/min	T	1.02 (1.81)	−4.22	1.13	4.68	1.68	0.04*
	C	0.11 (1.64)	−3.65	−0.14	3.57	2.35	
Sit-and-reach test, cm	T	3.81 (3.84)	−4.00	3.60	14.20	4.90	< 0.01*
	C	−1.19 (2.79)	−7.20	−1.30	4.70	3.60	
Jumping with legs apart, s	T	−0.01 (0.03)	−0.06	−0.01	0.06	0.04	0.55
	C	0.00 (0.04)	−0.09	−0.01	0.06	0.05	

Abbreviations: T, treatment group; C, control group; SD, standard deviation; IQR, interquartile range; reps, repetitions

^*^Statistically significant

To investigate the program effects on the subgroups, we conducted stratified analyses according to age and sex (Additional file 4, 5, 6 and 7). Muscle thickness (external oblique and left rectus femoris) increased in participants aged > 55 years and women. The effect of the exercise program on reducing BFP and waist circumference was distinctly shown in the group aged < 55 years, with a significant decrease in BFP in men and waist circumference in women. Moreover, women and participants aged < 55 years showed improved physical fitness. No distinct characteristics were found in biochemical data based on sex or age.

## Discussion

This study evaluated 8-week tailored program of aerobic and anaerobic exercises based on sex, age, physical activity level for middle-aged adults aged 40–69 years in South Korea. To ensure the effects of personalized exercise program, we used sonography, INBODY analysis, blood testing, and multiple physical fitness tests, and found the differences in the external oblique, left rectus femoris, BFP, waist circumference, cross-strait-up test, vertical jump, VO_2_ max, and sit-and-reach test results between treatment and control group.

Several studies demonstrated the effects of tailored program, which was consistent with our study. A 12-week personalized and community-based exercise program confirmed by heart rate reported fat loss and improved fitness metrics among 70 sedentary individuals compared with 72 controls [19]. In addition, personalized exercise programs were effective in participants with type 1 and postoperative complications [18], diabetes [20]. In addition, previous studies focused on a limited range of measurements [18–20, 36] or descriptive methods such as questionnaires and interviews [23, 24, 43], we validated the effects of tailored from multiple perspectives such as fitness metrics, image interpretation, and biochemical data.

Women experienced a stronger effectiveness of GPEP, whereas changes in GPEP varied according to age. Previous studies reported a significantly greater enhancement of the stretch-shortening cycle in older women than in similarly aged men [44]. In addition, when comparing concentric strength in women in their third and eighth decades, no difference in muscle quality was observed [45], whereas a decline in muscle quality was documented in men [46, 47]. These findings are consistent with our results. We also found a greater improvement in body composition and physical fitness in younger participants, whereas muscle thickness increased in the older age group. Previous studies reported the efficacy of guided exercise programs in elderly individuals aged 65 years [48, 49], which is inconsistent with our results.

The findings from this 8-week randomized controlled trial provide valuable insights into the effect of GPEP in improving not only physical fitness but also muscle thickness and body fat in adults aged 40–69 years. GPEP offered personalized exercise by finely adjusting exercise intensity based on individuals’ physical capacity measured using the GPAQ and by considering their age and sex. Notably, this study not only validated the effects of ultrasound-measured muscle thickness and reductions in body fat and waist circumference but also observed low dropout rates in the treatment group, with only three participants (9.1%) withdrawing—significantly below the typical 20% attrition rate [50]. Furthermore, this study distinguishes itself from previous research by incorporating hard data through self-reported surveys and ultrasound measurements. These measurements enable a more objective assessment of physical activity capacity and provide a robust basis for confirming statistical significance.

One limitation of this study is that the algorithm used to calculate the participants’ grades considered sex and age equally as factors influencing physical capacity. While both factors influence physical capacity, they do so in different ways and at different magnitudes [51]. Acknowledging this limitation, future research should explore more personalized approaches by incorporating a broader range of factors such as additional variables such as exercise compliance, co-morbidities, and motivation. to refine the grading system and enhance the applicability of GPEP. Another limitation of this study is that, although the core-focused circuit exercise program significantly increased abdominal muscle thickness in the treatment group, it was insufficient to account for the observed outcomes related to both rectus femoris muscles. One plausible explanation for the differing outcomes between the bilateral lower limbs is that, because most individuals are right-handed, the exercise may have aimed to correct asymmetry by further developing the left (nondominant) lower limb muscles to promote symmetry [52, 53]. Future research should rigorously examine the asymmetry of muscles in the bilateral lower extremities in relation to the participants’ dominant hands.

Despite significant improvements in core muscle, body fat, and physical fitness, GPEP did not lead to marked changes in blood test outcomes, likely due to the absence of dietary intervention and the relatively short study duration. Previous studies have generally employed longer intervention periods, averaging 16 weeks, which may have allowed for greater metabolic adaptations [18–21]. While our findings highlight meaningful short-term benefits, the lack of concurrent nutritional intervention and the study’s duration may have limited the overall impact on biochemical markers. Future research should investigate the combined effects of prolonged exercise programs and dietary interventions to better understand their synergistic influence on physical and metabolic health.

## Conclusions

This 8-week randomized controlled trial provides evidence that a GPAQ-based GPEP that was customized for middle-aged adults demonstrates significantly enhanced muscle thickness, reduced body fat, and improved physical fitness. The observed low dropout rate further indicates the feasibility and acceptability of the program among the participants, and thus underscores its potential for broader implementation as part of preventive health strategies. Our findings suggest that implementing a GPEP based on individual physical activity levels, age, and sex may contribute to improved physical health and potentially prevent sarcopenia in the middle-aged population. Further research is needed to evaluate the long-term effects of GPEP on overall metabolic health and its impact on middle-aged and older populations with adjusted weighting to accurately reflect the influence of age and sex on physical capacity. Additionally, exploring tailored adaptations based on specific musculoskeletal needs or physical conditions could enhance the program’s applicability and effectiveness for addressing age-related physical decline.

## Electronic supplementary material

Below is the link to the electronic supplementary material.

Additional file 1. Description of the physical fitness evaluations.
 Additional file 2. Muscle (abdominus and femoris) and fat measurements obtained via ultrasound.
 Additional file 3. Results of blood tests performed before and after exercise in the treatment and control groups.
 Additional file 4. Sex- and age-stratified differences in the ultrasound-quantified muscle thickness (external oblique and left rectus femoris) before and after exercise in treatment and control groups.
 Additional file 5. Sex- and age-stratified differences in body fat percentage and waist circumference before and after exercise in the treatment and control groups.
 Additional file 6. Sex- and age-stratified differences in physical fitness before and after exercise in the treatment and control groups.
 Additional file 7. Sex- and age-stratified differences in physical fitness indicators (monocyte, GPT, and ALP) before and after exercise in the treatment and control groups. Abbreviations: GPT, glutamic pyruvic transaminase; ALP, alkaline phosphatase.

## Data Availability

The data that support the findings of this study are available from the corresponding author, S. D. Y. upon reasonable request.

## Footnotes

1 Metabolic Equivalent; A method of indicating exercise intensity, where 1 MET represents the amount of oxygen required to maintain a resting state, and the oxygen consumption for various exercises and activities is expressed as a multiple of this value.

## Abbreviations

BFP: Body fat percentage
bpm: Beats per minute
BMI: Body mass index
C: Control group
GPAQ: Global Physical Activity Questionnaire
GPEP: GPAQ-based graded personalized exercise program
IQR: Interquartile range
LBM: Lean body mass
Lt: Left
Rt: Right
SD: Standard deviation
T: Treatment group
reps: Repetitions
