# Supplementary material for: A graded personalized exercise program’s effect on muscle and body fat: randomized controlled trial
Source: BMC Public Health. 2025 Apr 3;25:1258. doi: 10.1186/s12889-025-22453-5 (PMC11970005; doi:10.1186/s12889-025-22453-5)
Supplement: Supplementary file 1 — Additional file 1. Description of the physical fitness evaluations. Additional file 2. Muscle (abdominus and femoris) and fat measurements obtained via ultrasound. Additional file 3. Results of blood tests performed before and after exercise in the treatment and control groups. Additional file 4. Sex- and age-stratified differences in the ultrasound-quantified muscle thickness (external oblique and left rectus femoris) before and after exercise in treatment and control groups. Additional file 5. Sex- and age-stratified differences in body fat percentage and waist circumference before and after exercise in the treatment and control groups. Additional file 6. Sex- and age-stratified differences in physical fitness before and after exercise in the treatment and control groups. Additional file 7. Sex- and age-stratified differences in physical fitness indicators (monocyte, GPT, and ALP) before and after exercise in the treatment and control groups. Abbreviations: GPT, glutamic pyruvic transaminase; ALP, alkaline phosphatase. [file 12889_2025_22453_MOESM1_ESM.docx]

**Additional Files for**

**Effects of a Global physical activity questionnaire (GPAQ)-based Graded Personalized Exercise Program on Muscle and Body Fat: A Randomized Controlled Trial**

Jung Yeon Son ^1,2,¶^, Jiyun Jung ^3,¶^, Jung Eun Son ^1^, Sang Gyu Park^1^, Eo Jin Park ^1^, Junga Lee ^4^ and Seung Don Yoo ^1,5,^*

**Additional file 1. Description of the physical fitness evaluations**

***Cardiorespiratory Fitness (step box test, ml/kg/min)***

The equipment used for the test includes a custom-made step box (height: 30.5 cm), a metronome, a timer, a heartrate monitor, and a chair with a backrest. Participants were seated on a chair with a backrest and fitted with a heartrate monitor. After resting until their resting heartrate decreased to less than 100 bpm, they began stepping up and down the 30.5-cm step box to the beat of a 96-bpm metronome for 3 min following the start signal. After 3 min, the participants sat in the chair behind them and rested for 1 min; thereafter, their heartrates were recorded using a heartrate monitor.

Regression Model for Estimating VO_2_max during the Step Test:

Male VO_2_ max = 70.597 − 0.246 (Age) + 0.077 (Height) − 0.222 (Weight) − 0.147(1-min recovery heartrate)

Female VO_2_ max = 54.337 − 0.185 (Age) + 0.097 (Height) − 0.246 (Weight) − 0.122(1-min recovery heartrate)

***Muscle Strength (relative grip strength, %)***

The participants stood upright with their feet shoulder-width apart. Using a handgrip dynamometer (K-114, KL Sport, Republic of Korea), they gripped the handle with their second finger joint while keeping the measuring arm straight and maintaining a 15° angle between the arm and torso. The contralateral arm remained in a neutral position. Upon receiving the start signal, the participants applied maximal force and maintained their posture for 5 s. The test was conducted twice for each hand, alternating sides, for four trials. The highest recorded value was used to calculate and record the relative grip strength.

Relative Grip Strength (%) = [(Grip Strength (kg) / Body Weight (kg)] × 100

***Muscular Endurance (cross sit-up test, repetitions)***

The participants sat on a sit-up testing device (K-111, KL Sports, Republic of Korea) with their feet and knees secured. They lay back with their arms crossed in an "X" shape over their chest, placing their hands on their shoulders. The shoulder sensor was positioned at the midpoint of the C7 vertebra. On receiving the start signal, the participants raised their upper body until both elbows touched the top of their knees, ensuring that no momentum was used and that their hands remained on their shoulders. The number of successful repetitions within 1 min was recorded.

***Flexibility (sit-and-reach test, cm)***

The participants removed their shoes and sat with both feet flat against the front panel of a sit-and-reach testing device (K-115A; KL Sports, Seoul, Republic of Korea), while keeping their knees straight. They extended their arms forward with their fingertips touching the device, while ensuring that the hands remained aligned and that one hand did not extend farther than the other. At the start signal, the participants bent forward, pushed the device with their fingertips, and held the position for 3 s. The test was performed twice and the highest values were recorded.

***Agility (jumping with legs apart, s)***

The participants stood comfortably on the mat sensor of a vertical jump-testing device (K-119, KL Sports, Republic of Korea), with their feet naturally spaced apart. At the start signal, they spread both feet sideways as quickly as possible, while ensuring that both feet left the ground simultaneously. The time from when the feet left the ground to when they landed was recorded. The test was conducted twice and the highest results were obtained.

***Power (vertical jump, s)***

The participants stood on the mat sensor of a vertical jump-testing device (K-119, KL Sports, Korea). Using the momentum from their arms, bodies, and legs, they jumped as high as possible. The time from takeoff to landing on the sensor was measured. The test was conducted twice, and the best results were obtained.

**Additional file 2. Muscle (abdominis and femoris) and fat measurements obtained via ultrasound**

Measurements were taken bilaterally for the lower limbs, and only for the left side of the abdomen. Each site was measured three times and the mean value was recorded**.**

| *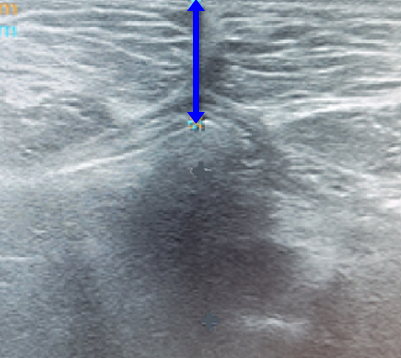* Total subcutaneous fat | The probe was positioned transversely 3 cm below the umbilicus, and the distance from the skin layer to the visible boundary was measured |
| --- | --- |
| *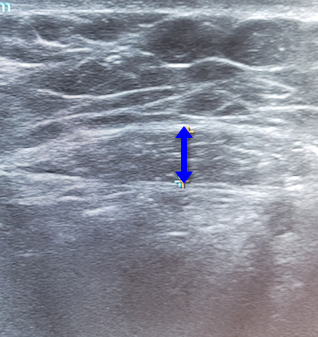*Rectus abdominis muscle | The probe was positioned transversely 4 cm to the left of the umbilicus, and the thickest portion of the muscle was measured. |
| *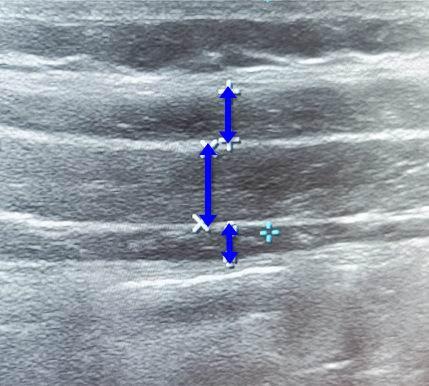*External oblique, internal oblique and transverse abdominis muscle | The probe was positioned vertically at the intersection of the umbilical line and an imaginary line connecting the left 12th costal cartilage curve and the anterior superior iliac spine (ASIS) for measurement |
| *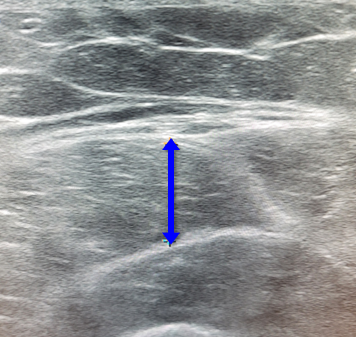*Rectus femoris muscle | The probe was positioned transversely at the midpoint between the superior border of the patella and the anterior superior iliac spine (ASIS), and the thickest portion of the muscle was measured |

**Additional file 3. Results of blood tests performed before and after exercise in the treatment and control groups.**

|  | **Group** | **Mean (SD)** | **Quantile** | | | | ***p*-value** |
| --- | --- | --- | --- | --- | --- | --- | --- |
|  |  |  | 0 | 50 | 100 | IQR |  |
| **ALP** | **T** | **−5.03 (9.23)** | **−24.00** | **−6.00** | **25.00** | **8.00** | **<0.01*** |
|  | **C** | **−0.48 (13.06)** | **−43.00** | **1.00** | **22.00** | **8.50** |  |
| ANC | T | 159.03 (741.50) | −904.00 | 128.00 | 2733.00 | 967.00 | 0.62 |
|  | C | 58.62 (890.12) | −2190.00 | 49.00 | 2292.00 | 702.50 |  |
| Basophil | T | 0.05 (0.29) | −0.60 | 0.00 | 0.60 | 0.30 | 0.37 |
|  | C | −0.02 (0.33) | −1.00 | 0.00 | 0.60 | 0.25 |  |
| BUN | T | −0.52 (3.12) | −6.00 | −1.00 | 7.00 | 5.00 | 0.86 |
|  | C | −0.65 (2.74) | −7.00 | 0.00 | 5.00 | 4.50 |  |
| CK | T | 69.42 (336.20) | −88.00 | 0.00 | 1895.00 | 49.00 | 0.89 |
|  | C | 0.65 (55.49) | −130.00 | 3.00 | 182.00 | 34.50 |  |
| Cr | T | 0.05 (0.14) | −0.14 | 0.04 | 0.66 | 0.13 | 0.89 |
|  | C | 0.04 (0.09) | −0.29 | 0.06 | 0.16 | 0.09 |  |
| CRP | T | −0.03 (0.11) | −0.54 | 0.00 | 0.10 | 0.04 | 0.59 |
|  | C | 0.01 (0.15) | −0.30 | 0.00 | 0.70 | 0.02 |  |
| Eosinophil | T | −0.13 (1.05) | −2.10 | −0.10 | 3.40 | 0.90 | 0.09 |
|  | C | 0.37 (1.18) | −1.10 | 0.20 | 5.00 | 1.05 |  |
| ESR | T | −1.00 (6.75) | −25.00 | −1.00 | 14.00 | 4.00 | 0.91 |
|  | C | −0.61 (7.74) | −19.00 | 0.00 | 20.00 | 6.00 |  |
| Ferritin | T | 13.53 (21.32) | −27.20 | 13.00 | 70.00 | 29.10 | 0.29 |
|  | C | 7.77 (21.80) | −34.00 | 7.00 | 60.00 | 20.10 |  |
| FI | T | 0.48 (3.00) | −8.00 | 0.10 | 7.80 | 3.00 | 0.40 |
|  | C | −0.12 (2.65) | −7.50 | −0.10 | 5.80 | 2.20 |  |
| FPG | T | 2.52 (8.76) | −8.00 | 2.00 | 28.00 | 11.00 | 0.55 |
|  | C | 0.68 (14.71) | −49.00 | 1.00 | 30.00 | 15.00 |  |
| GGT | T | 2.15 (6.22) | −7.00 | 0.00 | 24.00 | 4.00 | 0.10 |
|  | C | −2.58 (10.75) | −51.00 | −1.00 | 9.00 | 4.50 |  |
| GOT | T | 0.91 (5.99) | −7.00 | 0.00 | 29.00 | 4.00 | 0.86 |
|  | C | −0.16 (3.10) | −9.00 | 0.00 | 8.00 | 3.00 |  |
| **GPT** | **T** | **2.18 (4.88)** | **−9.00** | **2.00** | **13.00** | **5.00** | **0.02*** |
|  | **C** | **−0.48 (4.52)** | **−11.00** | **−1.00** | **10.00** | **6.00** |  |
| Hb | T | −0.08 (0.79) | −3.40 | 0.10 | 0.90 | 0.60 | 0.99 |
|  | C | 0.04 (0.52) | −0.80 | 0.00 | 1.30 | 0.80 |  |
| HbA1c | T | 0.05 (0.30) | −0.60 | 0.00 | 1.10 | 0.30 | 0.77 |
|  | C | 0.02 (0.27) | −1.00 | 0.10 | 0.40 | 0.20 |  |
| Hct | T | −0.55 (2.13) | −9.50 | −0.80 | 2.80 | 2.10 | 0.66 |
|  | C | −0.19 (1.62) | −3.30 | −0.40 | 3.80 | 1.95 |  |
| HDL | T | 1.97 (6.62) | −7.00 | 4.00 | 17.00 | 12.00 | 0.79 |
|  | C | 1.26 (6.27) | −11.00 | 1.00 | 16.00 | 8.50 |  |
| HOMA-IR | T | 0.13 (0.74) | −1.71 | −0.01 | 1.81 | 0.81 | 0.40 |
|  | C | −0.02 (0.66) | −1.23 | 0.03 | 1.43 | 0.90 |  |
| LDH | T | 4.09 (21.62) | −32.00 | 5.00 | 79.00 | 20.00 | 0.53 |
|  | C | 6.39 (19.61) | −32.00 | 3.00 | 78.00 | 13.00 |  |
| LDL | T | 7.97 (23.76) | −28.00 | 6.00 | 85.00 | 21.00 | 0.76 |
|  | C | 4.71 (17.97) | −59.00 | 6.00 | 32.00 | 18.00 |  |
| Lymphocyte | T | −1.41 (8.04) | −30.80 | 0.00 | 10.10 | 7.90 | 0.85 |
|  | C | −0.93 (6.70) | −13.80 | −0.60 | 11.20 | 10.20 |  |
| MCH | T | 0.04 (1.12) | −5.90 | 0.20 | 1.10 | 0.50 | 0.24 |
|  | C | 0.11 (0.42) | −0.80 | 0.10 | 1.20 | 0.45 |  |
| MCHC | T | 0.36 (0.49) | −1.24 | 0.36 | 0.98 | 0.68 | 0.18 |
|  | C | 0.24 (0.38) | −0.54 | 0.30 | 1.12 | 0.51 |  |
| MCV | T | −0.89 (2.79) | −15.60 | −0.40 | 1.90 | 0.90 | 0.79 |
|  | C | −0.41 (0.93) | −1.40 | −0.60 | 3.00 | 0.75 |  |
| **Monocyte** | **T** | **−0.45 (1.42)** | **−6.30** | **−0.40** | **2.10** | **1.20** | **0.03*** |
|  | **C** | **0.15 (1.46)** | **−4.00** | **0.10** | **2.40** | **1.35** |  |
| MPV | T | 0.08 (0.37) | −0.70 | 0.00 | 1.00 | 0.30 | 0.37 |
|  | C | −0.01 (0.41) | −0.80 | −0.10 | 0.80 | 0.55 |  |
| Neutrophil segment | T | 1.91 (8.05) | −9.50 | 0.80 | 33.80 | 7.90 | 0.70 |
|  | C | 0.46 (6.61) | −11.10 | 0.20 | 12.60 | 8.50 |  |
| PLT | T | −0.06 (33.98) | −68.00 | −1.00 | 101.00 | 34.00 | 0.64 |
|  | C | −2.48 (34.74) | −147.00 | 1.00 | 39.00 | 33.00 |  |
| RBC | T | −0.02 (0.16) | −0.39 | −0.04 | 0.30 | 0.21 | 0.72 |
|  | C | 0.00 (0.17) | −0.31 | −0.01 | 0.49 | 0.26 |  |
| TC | T | 7.94 (21.57) | −23.00 | 6.00 | 84.00 | 23.00 | 0.28 |
|  | C | 11.84 (29.52) | −65.00 | 11.00 | 122.00 | 23.50 |  |
| TG | T | −0.73 (28.60) | −54.00 | 3.00 | 45.00 | 43.00 | 0.39 |
|  | C | −9.97 (53.03) | −121.00 | −11.00 | 107.00 | 66.00 |  |
| Uric acid | T | 0.21 (0.62) | −1.00 | 0.20 | 1.30 | 0.90 | 0.74 |
|  | C | 0.15 (0.80) | −2.00 | 0.30 | 1.40 | 1.05 |  |
| WBC | T | 0.08 (0.76) | −1.44 | −0.10 | 1.82 | 1.08 | 0.78 |
|  | C | 0.01 (1.16) | −2.27 | −0.03 | 3.15 | 1.34 |  |
| SBP | T | 4.24 (11.93) | −22.00 | 4.00 | 26.00 | 13.00 | 0.78 |
|  | C | 3.32 (14.65) | −26.00 | 6.00 | 31.00 | 14.00 |  |
| DBP | T | 3.27 (10.73) | −19.00 | 3.00 | 26.00 | 11.00 | 0.33 |
|  | C | 0.87 (8.55) | −15.00 | 0.00 | 20.00 | 11.00 |  |

Abbreviations: T, treatment group; C, control group; SD, standard deviation; IQR, interquartile range; ALP, alkaline phosphatase; ANC, absolute neutrophil count; BUN, blood urea nitrogen; CK, creatine kinase; Cr, creatine; CRP, C-reactive protein; ESR, erythrocyte sedimentation rate; FI, fasting insulin; FPG, fasting plasma glucose; GGT, gamma-glutamyl transferase; GOT, glutamic oxaloacetic transaminase; GPT, glutamic pyruvic transaminase; Hb, hemoglobin; HbA1c, glycated hemoglobin; HCT, hematocrit; HDL, high density lipoprotein; HOMA-IR, homeostasis model assessment of insulin resistance; LDH, lactate dehydrogenase; LDL, low density lipoprotein; MCH, mean cell hemoglobin; MCHC, mean cell hemoglobin concentration; MCV, mean cell volume; MPV, mean platelet volume; PLT, platelet; RBC, red blood cell; TC, total cholesterol; TG, triglyceride; WBC, white blood cell; SBP, systolic blood pressure; DBP, diastolic blood pressure.


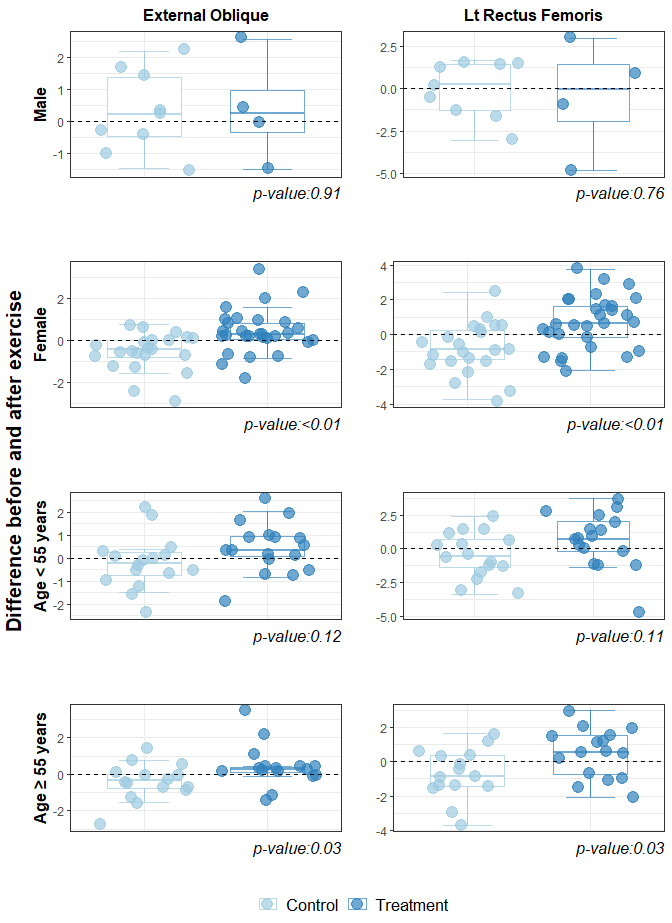


**Additional file 4**. Sex- and age-stratified differences in the ultrasound-quantified muscle thickness (external oblique and left rectus femoris) before and after exercise in treatment and control groups.


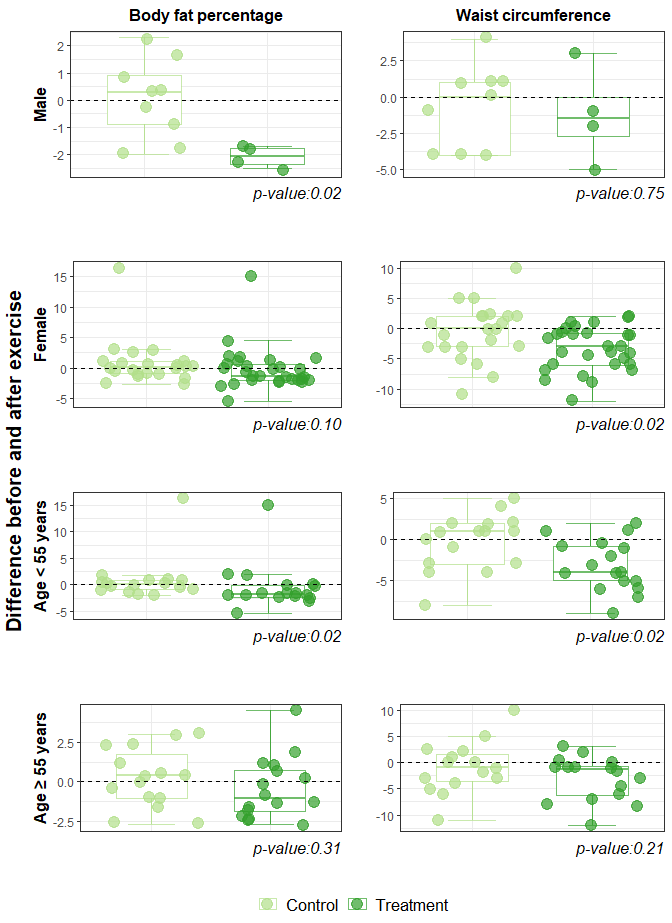


**Additional file 5**. Sex- and age-stratified differences in body fat percentage and waist circumference before and after exercise in the treatment and control groups.


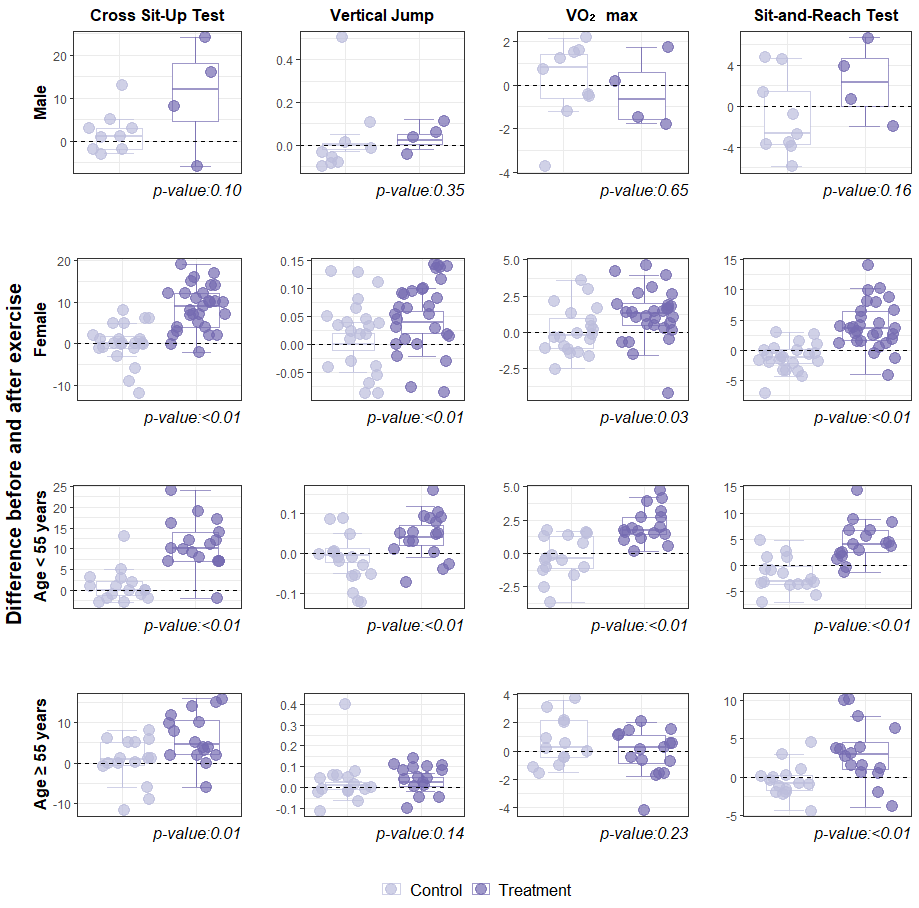


**Additional file 6.** Sex- and age-stratified differences in physical fitness before and after exercise in the treatment and control groups.


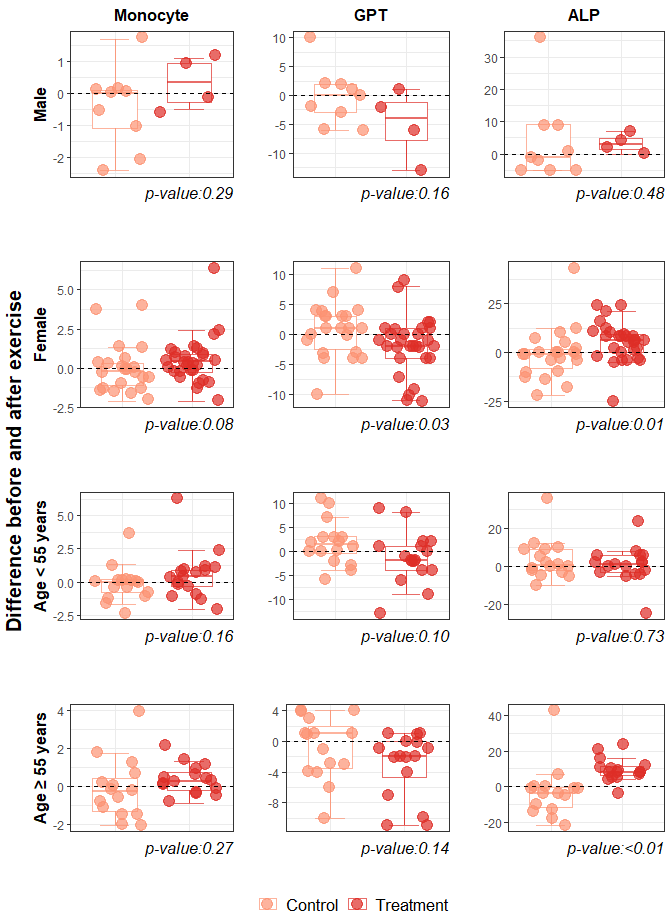


**Additional file 7**. Sex- and age-stratified differences in physical fitness indicators (monocyte, GPT, and ALP) before and after exercise in the treatment and control groups. Abbreviations: GPT, glutamic pyruvic transaminase; ALP, alkaline phosphatase
